# Effect of Thermal Cycle on Microstructure Evolution and Mechanical Properties of Selective Laser Melted Low-Alloy Steel

**DOI:** 10.3390/ma12213625

**Published:** 2019-11-04

**Authors:** Xueliang Kang, Shiyun Dong, Hongbin Wang, Shixing Yan, Xiaoting Liu, Huiping Ren

**Affiliations:** 1 School of Materials Science and Engineering, Shanghai University, Shanghai 200444, China; kangxueliangyx@163.com; 2 National Key Laboratory for Remanufacturing, Army Academy of Armored Forces, Beijing 100072, China; ysxneu@163.com (S.Y.); liuxtbit@126.com (X.L.); 3 Key Laboratory of Integrated Exploitation of Bayan Obo Multi-Metal Resources, Inner Mongolia University of Science and Technology, Baotou 014010, China; renhuiping@sina.com

**Keywords:** selective laser melting, thermal cycle, microstructure evolution, mechanical property, low-alloy steel

## Abstract

Low-alloy steel samples were successfully fabricated by selective laser melting (SLM). The evolution of the microstructure and the mechanical properties were investigated with different values of the energy area density (EAD). The results revealed that the initial solidification microstructures of the single tracks with different EADs were all martensite. However, the microstructures of bulk samples under different EADs were not martensite and differed significantly even from one another. When EAD increased from 47 to 142 J/mm^2^, the mixed lower bainite and martensite austenite microstructure changed to granular bainite; further, the morphology of bainite ferrite gradually changed from lath to multilateral. Moreover, with the increase of EAD, the grain size was remarkably reduced because of the increasing austenitizing periods and temperature during thermal cycling. The average grain size was 1.56 µm, 3.98 µm, and 6.31 µm with EADs of 142 J/mm^2^, 71 J/mm^2^, and 47 J/mm^2^, respectively. Yield strength and tensile strength of the SLM low-alloy steel increased with the increase in EAD; these values were significantly more than those of the alloys prepared by traditional methods. The microstructure of the SLM low-alloy steel samples is not uniform, and the inhomogeneity becomes more significant as EAD decreases. Simultaneously, when EAD decreases, the fracture mechanism changes from ductile to a mixture of ductile and brittle fracture; this is in contrast to the samples prepared by traditional methods. This study also found a stress concentration mechanism around large pores during plastic deformation that resulted in a brittle fracture. This indicates that large-sized pores significantly degrade the mechanical properties of the specimens.

## 1. Introduction

Selective laser melting (SLM) is a manufacturing technique rapidly developing worldwide because of its ability to deliver metal parts with high density, good surface quality, and high mechanical properties [1,2,3]. During the SLM process, the computer-aided design (CAD) model of the part is computationally sliced in thin layers. According to pre-set parameters (such as laser power, scanning speed, scanning path, etc.), the pre-laid powders are melted and then solidified by moving the laser beam. After printing one layer, the process is repeated until the SLM component is completed. The SLM technology has obvious advantages over traditional methods in manufacturing complex structural components, and for this reason it is increasingly being used in industry.

Recently, the SLM technology has been widely used to fabricate metal components, including parts of titanium alloys [4,5,6], copper alloys [7,8], aluminum alloys [9], nickel-based alloys [10], etc. Several studies reported on the preparation of ferrous alloys by SLM, among which many focused on 316L steel [11,12,13]. For instance, Tomasz Kurzynowski et al. [14] studied the correlation between SLM pre-set parameters and the final properties of 316L stainless steel, including its microstructure. They found that the laser energy density and the scanning strategy strongly affect the austenite cellular substructure and the amount of ferrite, as well as the texture. Almangour et al. [15] mainly focused on the microstructural arrangement and size of TiC nanoparticles under different processing parameters, and on the grain sizes and tribological performances of SLM-processed nanocomposite parts. Di et al. [16] studied the effect of laser speed on the grain structure development of 316L stainless steel, including grain growth mode and their final density, and on its mechanical properties.

The matrix phase of 316L steel is austenite, which does not change under the effect of thermal cycling. However, few studies investigated the effect of thermal cycling on the microstructure of 316L steel. The microstructure of low-alloy steel is very sensitive to the influence of heat. After solidification, austenite, pearlite, upper, granular and lower bainite, and martensite may form under different cooling conditions. These microstructures also undergo phase transformation when heated. Tempered martensite, recrystallized ferrite, tempered bainite, tempered toxote troostite, and tempered sorbite may form at different heating temperatures. The different microstructures have a great impact on the mechanical properties of the components.

Several studies are available on low-alloy steel. Yue et al. [17] studied the evolution of bainite and the mechanical properties of direct laser deposited 12CrNi2 alloy steel for different laser power. They found that the microstructures changed under different processing parameters, though they did not unravel their formation mechanism. Mingwei et al. [18] investigated the microstructural evolution of 24CrNiMo steel for different SLM power and found that heat accumulation occurs during SLM. However, the authors did not analyze the microstructure formation mechanism. Zuo et al. [19] studied the microstructure evolution of 24CrNiMoY alloy steel parts in SLM. They found different microstructures in at least one sample, and while they probed thermal cycling in the SLM process, they did not unravel its effect on the microstructure evolution.

A SLM part is fabricated track by track and layer by layer. As a consequence, the temperature field changes dramatically during the process [20,21]. Heat input and heat accumulation can have a significant impact on the preformed microstructure, so the final microstructure of SLM-formed low-alloy steel can be very complex. The thermal cycling process is different for the specimens formed under different SLM parameters, and so is the phase transformation. Thus, different microstructures are finally obtained. At present, limited research has focused on the processing parameters, the effect of thermal cycle, the microstructure evolution, and the mechanical properties of low-alloy steel in SLM. In this paper, we discuss in detail the effect of energy area density (EAD), as a substitution to scanning speed, on these properties.

## 2. Experimental Materials and Methods

### 2.1. Powder Material

**Figure 1:**
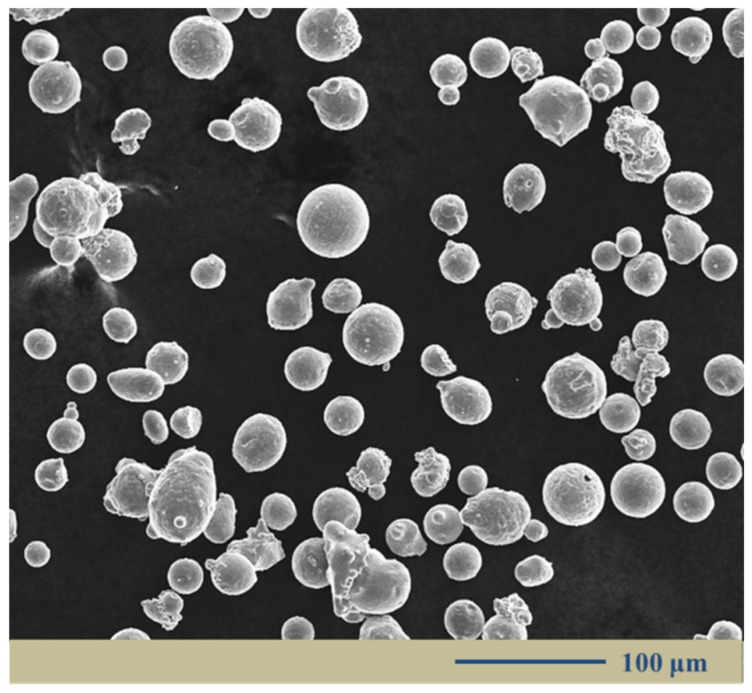
Scanning electron microscopy (SEM) image of low-alloy steel powder.

The low-alloy steel powder was prepared by vacuum induction melting gas atomization method. The average diameter of the powder grains was below 50 µm and their shapes were nearly spherical. A representative image of the powder morphology is shown in Figure 1. The chemical compositions are listed in Table 1.

### 2.2. Microstructural Characterization and Mechanical Test

**Figure 2:**
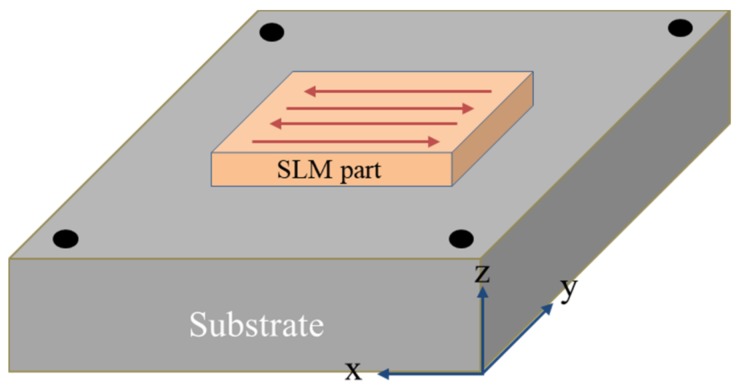
Schematic illustration of a selective laser melting (SLM)-fabricated low-alloy steel part.

**Figure 3:**
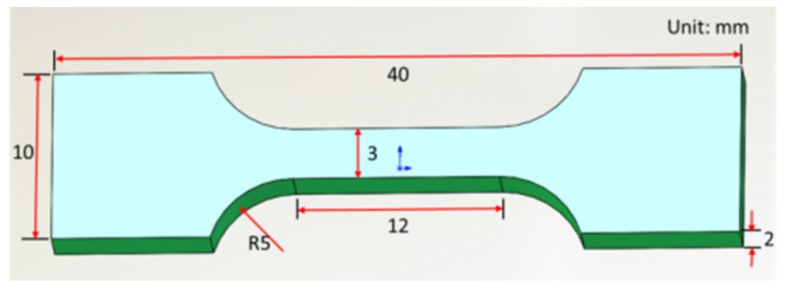
Dimensions of the tensile plate specimens.

The as-fabricated samples were microstructurally characterized at their yz cross section. The samples were grounded and polished following standard procedures and were etched in alcohol nitrate solution (5 mL HNO_3_ + 95 mL C_2_H_5_OH) for about 10 s prior to microstructural characterization. This was carried out using a PMG3 optical microscope (OM) and a Nova NanoSEM50 field emission scanning electron microscope (FESEM) at 30 kV. The grain morphology and size were obtained by electron backscatter diffraction (EBSD) characterization. The samples were electropolished (8 mL HClO_4_ + 92 mL C_2_H_5_OH, −15 °C, 15 s) prior to EBSD analysis. We characterized the substructures of the samples by transmission electron microscopy (TEM) and measured their phase transition temperature using a thermal expansion tester with a heating rate of 10 °C/s and a cooling rate of 5 °C/s. The tensile test was carried out at room temperature on a CMT5105 testing machine at a stretching speed of 0.6 mm/min. We used scanning electron microscopy (SEM) to characterize the tensile fracture surfaces. The tensile plate specimens were cut along the x direction as depicted in Figure 2, and their dimensions are reproduced in Figure 3.

### 2.3. SLM Process

The SLM samples were prepared in a Renishaw AM400 system. The powder was dried at 90 °C for 2 h prior to its usage and the oxygen content in the fabrication cabin was kept below 200 ppm. The diameter of the laser spot was 70 µm. A zigzag scanning strategy was used (Figure 2) with the overlap ratio between adjacent tracks set at 45%. Detailed working parameters are listed in Table 2.

**Figure 4:**
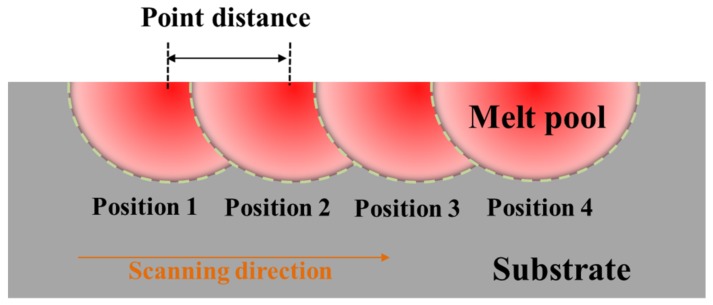
Schematic illustration of point distance of the pulsed laser.

Several sample batches were prepared, using different scanning speeds. It should be noted that the laser was pulsed and the laser spot scanning speed (v) was determined by point distance (d) and exposure time (t) through the relation [22,23]:

(1) $$v=\frac{d}{t}$$

where d represents the distance between adjacent positions and t represents the residence time of the laser spot in one position. The laser scanning mode and the point distance are schematically represented in Figure 4.

The energy area density (EAD) is used to measure the input laser energy and is defined as [3]:

(2) $$\mathrm{EAD}=\frac{P}{v*D}$$

where P is the laser power (W), v is the scan speed (mm/s), and D is the laser spot diameter (mm).

We used the finite element method to study the evolution of the temperature field. We experimentally measured the thermal physical properties of the fabricated low-alloy steel below 900 °C and used the dynamic simulation software JMATPRO to calculate them above this temperature, due to the testing limitations. The thermal physical properties evolution is shown in Table 3.

## 3. Results and Discussion

### 3.1. Formability Characterization

**Figure 5:**
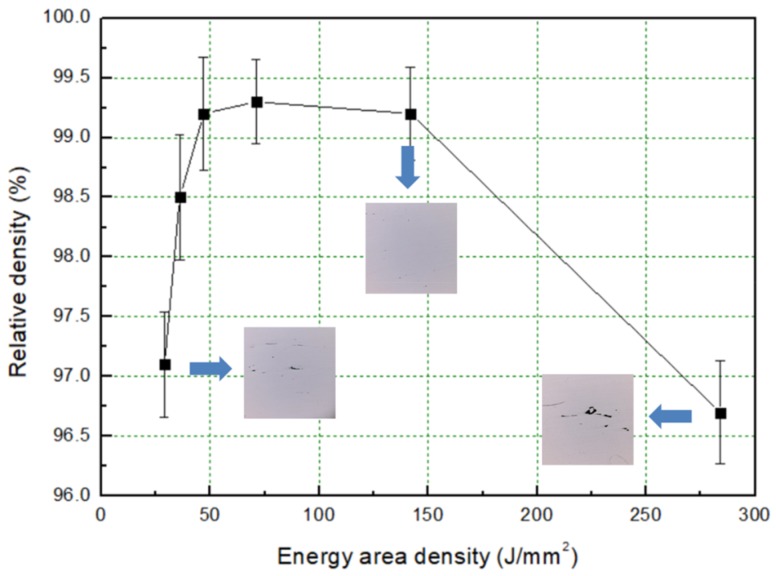
Evolution of the relative density of SLM low-alloy steel with EAD.

The SLM block formability is a key factor for its mechanical properties [24,25]. The formability can be controlled by optimizing the process parameters, and EAD is a crucial one. Figure 5 shows the evolution of the SLM parts relative density with an increasing EAD. To measure the relative density, we used the Archimedes principle. As seen from Figure 5, the density evolution is not monotonic. For an EAD in the 47–142 J/mm^2^ range, the relative density of the samples reaches up to 99%, so that the metallographic section of the sample displays a reduced porosity. When EAD lays below 50 J/mm^2^ or exceeds 150 J/mm^2^, the relative density decreases due to the macro-defects seen from the reported cross sections.

### 3.2. Microstructural Characterization

**Figure 6:**
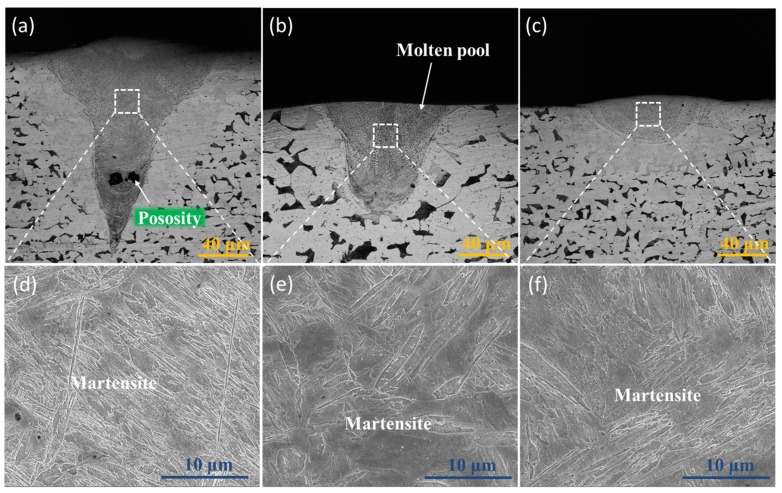
Molten pool morphologies and corresponding microstructural SEM images of SLM low-alloy steel samples grown with different EADs: (**a**,**d**) EAD = 284 J/mm^2^; (**b**,**e**) EAD = 71 J/mm^2^; (**c**,**f**) EAD = 29 J/mm^2^.

**Figure 7:**
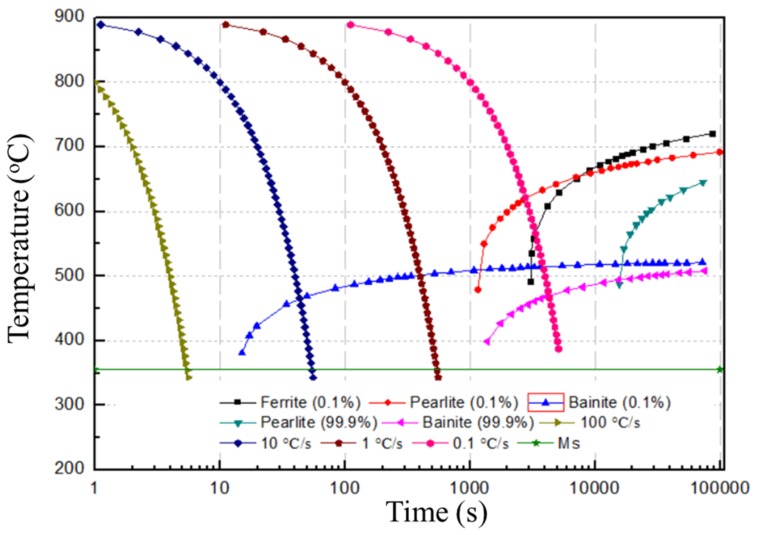
Continuous cooling transition (CCT) curve of low-alloy steel.

Due to rapid cooling and solidification, the SLM samples have a non-equilibrium microstructure [26], and as in all alloys with a solid phase, this will change during the thermal cycle. Thus, a complex microstructure results in these SLM-fabricated alloys [27,28]. Figure 6 shows the cross-section morphology and the corresponding microstructure of the SLM single tracks at different EADs. The microstructures of the three samples are martensite, as seen from Figure 6d–f. This is because the single-track microstructure is only imposed by the cooling rate of the SLM molten pool. The cooling rate ∆T/∆t can be calculated by [29]:

(3) $$\frac{∆T}{∆t}=\frac{\alpha_{\lambda }Q\sqrt{v}}{d_{m}^{2}\sqrt{2\rho \mathrm{ckd}}}$$

where α_λ_ is the absorptivity at laser wavelength λ, Q is the laser power, v is the scanning speed, ρ is the metal density, c is the specific heat, k is the thermal conductivity, and *d_m_* is the diameter of the melt pool. According to Equation (3), the cooling rate of the molten pool can reach values of 10^5^–10^6^ °C/s during the solidification process. From the continuous cooling transition (CCT) curve of SLM low-alloy steel shown in Figure 7, it can be seen that when the cooling rate of the molten pool is greater than 100 °C/s, the microstructure at room temperature mainly consists of martensite. Since the cooling rate of the molten pool during the SLM process is far greater than 100 °C/s, the single track microstructure is the same after cooling regardless of the EAD, mainly consisting of martensite as shown in Figure 6d–f.

**Figure 8:**
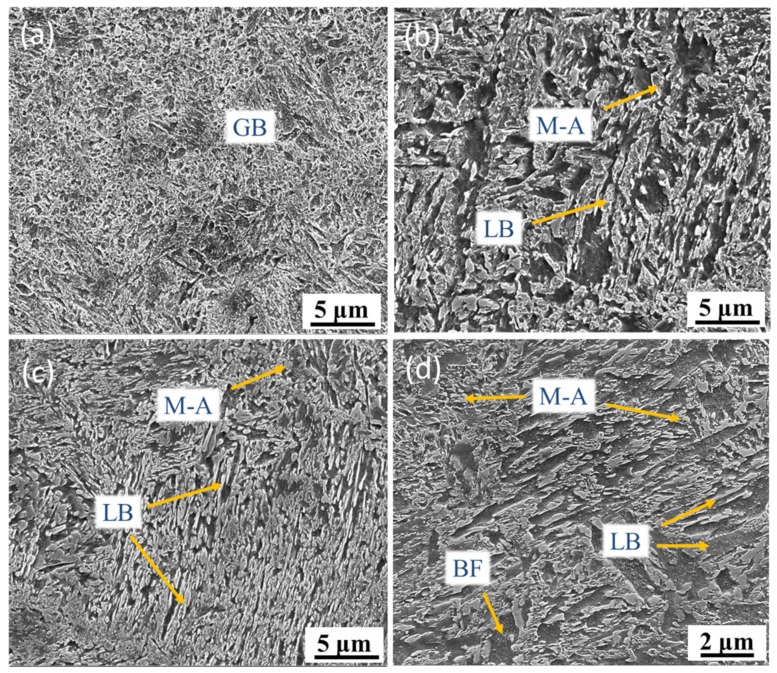
Microstructures of SLM low-alloy steel samples grown with different EADs: (**a**) EAD = 142 J/mm^2^; (**b**) EAD = 71 J/mm^2^; (**c**) EAD = 47 J/mm^2^; and (**d**) zoomed-in image of (**b**). GB: granular bainite; M-A: martensite–austenite; LB: lower bainite; BF: bainitic ferrite.

However, the bulk samples microstructure is no longer martensite, and samples manufactured with different EADs have different microstructures, as shown in Figure 8. At a high EAD (142 J/mm^2^), the microstructure is granular bainite (GB) composed of bainitic ferrite (BF) and martensite–austenite (M-A) constituents. As EAD reaches 71 J/mm^2^, the GB transforms into lower bainite (LB), which significantly increases in content as EAD further decreases to 47 J/mm^2^.

**Figure 9:**
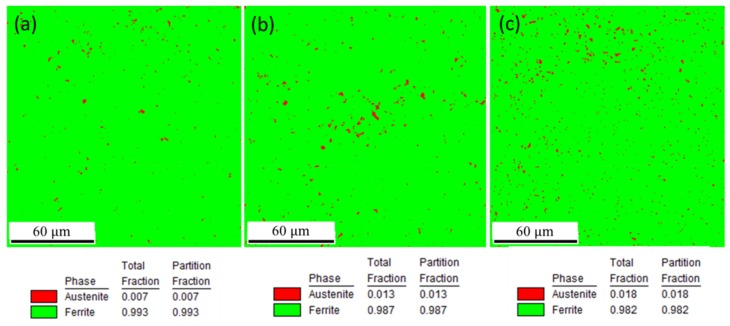
Phase composition of SLM low-alloy steel samples grown with different EADs: (**a**) EAD = 142 J/mm^2^; (**b**) EAD = 71 J/mm^2^; and (**c**) EAD = 47 J/mm^2^.

According to the equilibrium phase diagram of low-alloy steel, the microstructure at room temperature is mainly composed of ferrite and cementite. Austenite is also present under rapid solidification conditions. The phase content and distribution was studied by EBSD, as shown in Figure 9. As seen, retained austenite is found in all samples, and its content increases with the decrease of EAD. The presence of retained austenite is caused by the high cooling rate, and its content increases with it.

**Figure 10:**
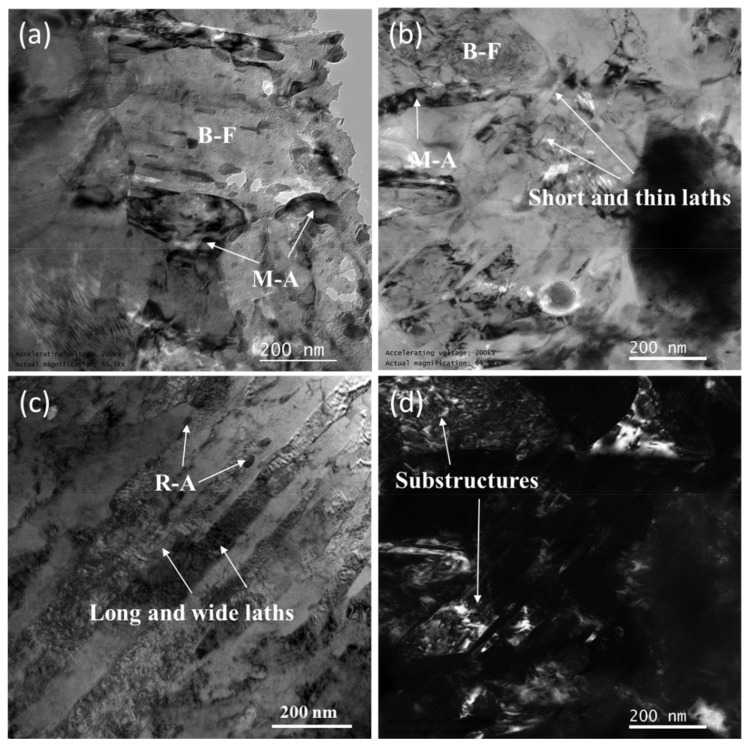
TEM morphology of SLM low-alloy steel samples grown with different EADs: (**a**) EAD = 142 J/mm^2^; (**b**) EAD = 71 J/mm^2^; (**c**) EAD = 47 J/mm^2^; and (**d**) dark field image of (**b**). R-A: retained austenite.

We used TEM to characterize the samples’ substructure further, and the results are reported in Figure 10. As can be seen, at a low EAD (47 J/mm^2^), the morphology of ferrite is that of parallel laths of about 100 nm in width, with retained austenite (RA) in between. With the increase of EAD (71 J/mm^2^), the lath-like ferrite was refined to a width of about 30 nm only. In addition, complex substructures of polygonal BF are formed. When EAD reaches 142 J/mm^2^, the sample microstructure consists of mainly polygonal BF and fine M-A. From the substructure characterization of the samples, it can be concluded that the microstructure average size is reduced by increasing EAD.

**Figure 11:**
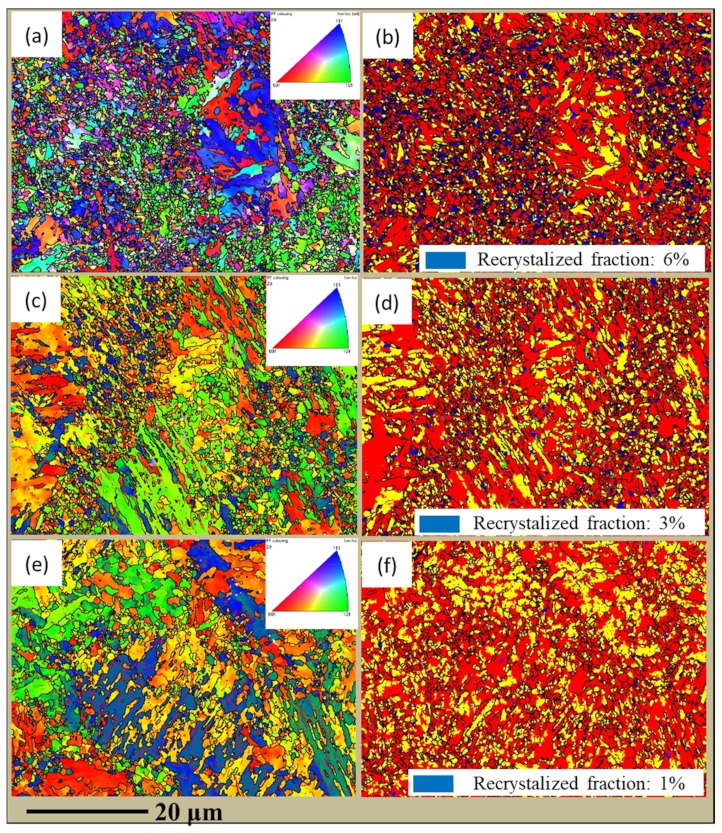
Inverse pole figures and recrystallized grains distribution of SLM low-alloy steel samples grown with different EADs: (**a**,**b**) EAD = 142 J/mm^2^; (**c**,**d**) EAD = 71 J/mm^2^; (**e**,**f**) EAD = 47 J/mm^2^.

In order to determine the grain size of SLM low-alloy steel samples at different EADs, we carried out EBSD analysis. According to the inverse pole figures (IPFs) of Figure 11, the differences in grain size among the samples is significant, with average values of 1.56 µm, 3.98 µm, and 6.31 µm at EADs of 142 J/mm^2^, 71 J/mm^2^, and 47 J/mm^2^, respectively. As expected from the grain size decreasing with the increase of EAD, the morphology of the grains changed from lath-like to multilateral.

**Figure 12:**
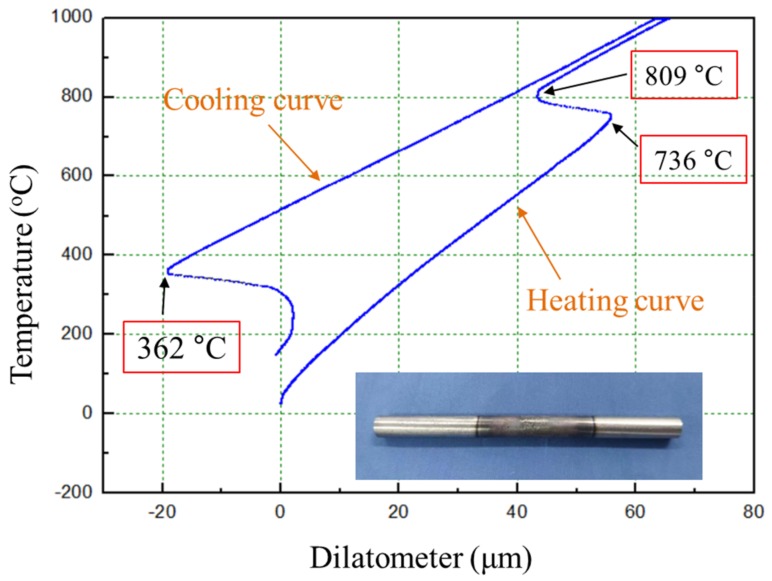
Thermal expansion curve during heating and cooling of the low-alloy steel.

As discussed above, the as-solidified molten pool microstructure does not vary with EAD, and the difference in bulk sample microstructure and grain size is mainly due to the thermal cycle, which causes martensite to undergo different phase transformations. The martensite starting temperature (Ms) in the low-alloy steel was measured at 362 °C (Figure 12), which is very close to the value of 382.5 °C obtained from the equation [30]:

(4) $$\begin{array}{ll} M_{s}\left(℃\right)=539 & -423\left(\%C\right)-30.4\left(Mn\right)-17.7\left(\%Ni\right)-12.1\left(\%Cr\right)\\ & -7.5\left(\%\mathrm{Mo}\right) \end{array}$$

Similarly, the temperatures AC_1_ and AC_3_ were measured to be 736 °C and 809 °C, respectively. However, the bainite start temperature (Bs) cannot be determined from the measured curve of Figure 12, though its value can be calculated to be about 520 °C by:

(5) $$\begin{array}{ll} B_{s}\left(℃\right)=830 & -270\left(\%C\right)-90\left(Mn\right)-37\left(\%Ni\right)-70\left(\%Cr\right)\\ & -83\left(\%\mathrm{Mo}\right) \end{array}$$

**Figure 13:**
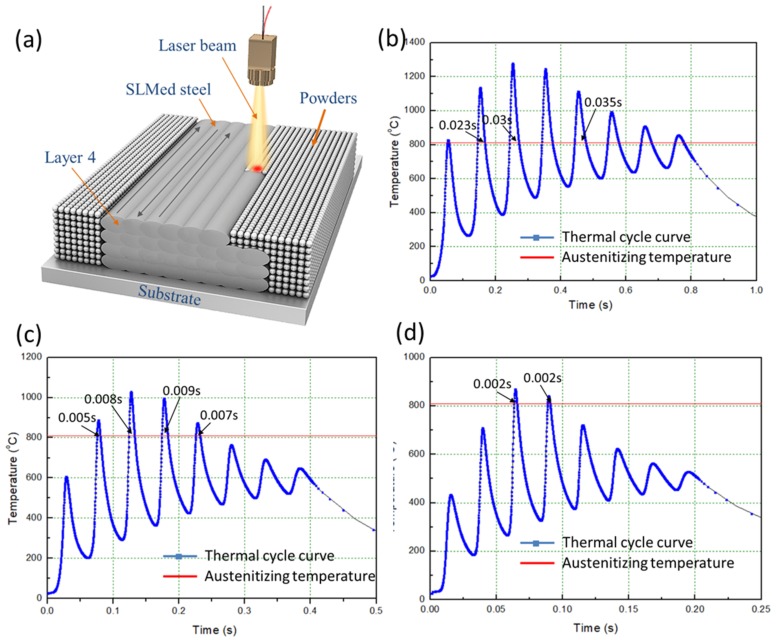
(**a**) Schematic diagram of SLM formation of low-alloy steel; (**b**–**d**) thermal cycle curves in the middle point of track three, layer three, at different EADs: (**b**) EAD = 142 J/mm^2^; (**c**) EAD = 71 J/mm^2^; (**d**) EAD = 47 J/mm^2^.

To study the effect of thermal cycling on the microstructure evolution, we simulated a temperature field of the block sample (four layers, eight tracks per layer) depicted in Figure 13a. Figure 13b–d shows the influence of the heat from the fourth layer on the temperature at a fixed position (middle point of track three, layer three, labeled A) for different EAD. It can be seen that point A undergoes several heating and cooling cycles during the fabrication of layer four, with significantly different heating temperature and time. The specific parameters of the thermal cycle process are reported in Table 4.

As the temperature raises to AC_3_, the martensite entirely transforms into austenite. Austenitization is a continuous process of nucleation and growth. The nucleation rate I can be calculated by

(6) $$I=C\times \exp\left(-\frac{Q+W}{\mathrm{kT}}\right)$$

where C is a constant, Q is the diffusion activation energy, W is the critical nucleation energy of the crystal, k is the Boltzmann constant, and T is the temperature. According to Equation (6), a higher temperature enhances the nucleation rate and consequently the grain size decreases. As can be seen from Table 4, the maximum heating temperature at point A increases with EAD, resulting in a higher nucleation rate and a finer grain size. As evidenced in Figure 11b,d,f, our results consistently support this behavior for the nucleation rate (recrystallized fraction) of austenite.

Furthermore, it is known that multiple austenitizing processes can contribute to grain refinement [31,32]. As seen from Table 4, the microstructure was austenitized 2, 4, and 7 times for increasing EAD, so an increasingly refined grain size is expected.

**Figure 14:**
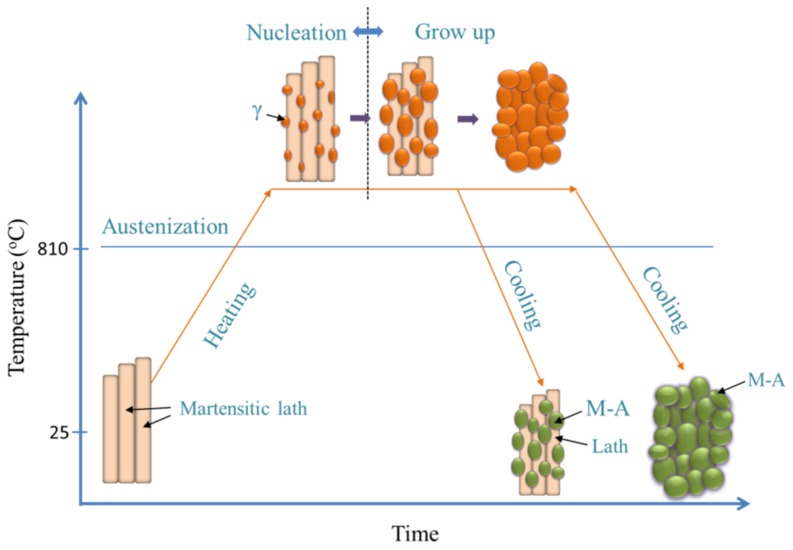
Schematic illustration of the microstructure evolution process.

In addition to the size, the morphology of the grains also changes. Austenite grains nucleate at boundaries of martensitic laths during the austenitizing process. The nucleation and growth of the grains require a long time because they depend on the components’ diffusion. If the austenitizing process is too short, the components diffusion is not uniform and only limited austenite grains form, thus an untransformed lath-like structure is retained during subsequent cooling. If the components are provided with sufficient diffusion times, the multilateral austenite grains completely replace the lath-like structure and retain their morphology upon cooling. The process is schematically represented in Figure 14.

As seen from Table 4, the sample fabricated at an EAD of 142 J/mm^2^ experienced a high temperature and a quite long heating time. Therefore, the components diffusion is enhanced, and the grains of the high-EAD samples are expected to be mostly multilateral. Instead, the grains of the low-EAD sample mostly display a lath-like morphology.

### 3.3. Tensile Tests Analysis

The yield stress (YS), the ultimate tensile stress (UTS), and the elongation (EL) of the samples are listed in Table 5. Here, the mechanical properties of the low-alloy steel prepared by traditional methods (labeled “Sample A”) that we tested are also listed. For EADs between 47 and 142 J/mm^2^, the yield strength and ultimate tensile strength increases with the EAD of the SLM low-alloy steels. However, the behavior of elongation with EAD is not obvious. It is worth mentioning that YS and UTS of the SLM low-alloy steel prepared in this paper can reach 1256 MPa and 1428 MPa, respectively, far exceeding those of the alloys prepared by traditional methods and those reported in literature for alloy steel. However, in samples with EAD of 36 J/mm^2^, the mechanical properties are significantly reduced.

The presence of many substructures in the microstructure is an important reason for the outstanding mechanical properties of alloy steels, as shown in Figure 10. These substructures can effectively hinder the movement of dislocations. Furthermore, crack propagation can be prevented by the diffuse distribution of M-A in the matrix, thus improving the steel strength.

The grain size is also a key factor in the strength of the specimen. The effect of grain size on yield strength can be calculated through the Hall–Petch relation [33]

(7) $$\sigma_{y}=\sigma_{0}+kd_{g}{}^{-0.5}$$

where $\sigma_{y}$ is the yield strength, *d_g_* is the average grain size, $\sigma_{0}$ and *k* are material constants.

As discussed, the average grain size is 1.56 µm, 3.98 µm, and 6.31 µm for samples with EADs of 142 J/mm^2^, 71 J/mm^2^, and 47 J/mm^2^, respectively, thus much smaller than that prepared by traditional methods. According to Equation (7), refined grains increase the yield strength and improve the plasticity of the sample.

### 3.4. Fracture Analysis

**Figure 15:**
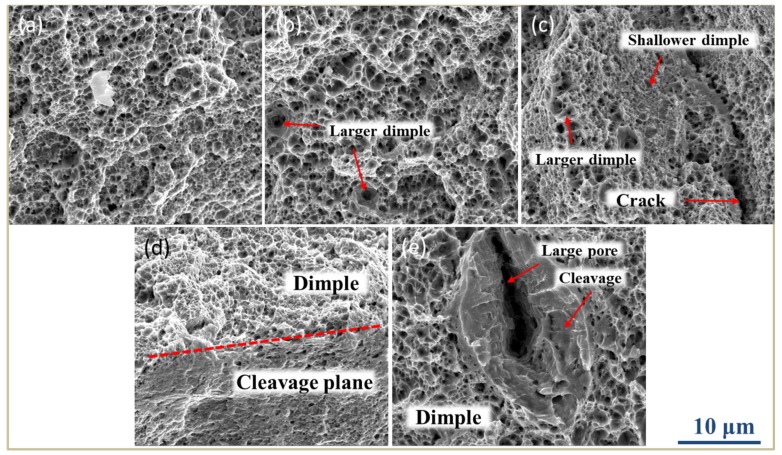
SEM images of fractures in SLM low-alloy steel samples grown with different EADs: (**a**) EAD = 142 J/mm^2^; (**b**) EAD = 71 J/mm^2^; (**c**,**d**) EAD = 47 J/mm^2^, and (**e**) EAD = 36 J/mm^2^.

The SEM fracture images of SLM low-alloy steel samples grown with different EADs are shown in Figure 15. For the three samples, the fracture mechanism was ductile. Overall, when EAD is highest (EAD = 142 J/mm^2^), the dimples are smaller because the grains are finer. Some smaller pores are seen in the fracture of Figure 15b. The pore size is nearly equal that of the dimples, and little influence on the surrounding dimples can be derived. Such micro-size pores can hardly be avoided in SLM processes. However, we can infer that their effect on the tensile properties of the samples is minimal.

The most important feature of the SLM low-alloy steel samples is the uneven fracture mechanism. One of the consequences of the non-uniform fracture morphologies is that different fracture properties appear in the cross section images. As shown in Figure 15c, different types of fracture properties are found in one area. Furthermore, as shown in Figure 15d, the dimples corresponding to ductile fracture and the smooth cleavage surfaces corresponding to brittle fracture occur simultaneously. Such a peculiar phenomenon is caused by the inhomogeneity of the microstructure. As seen from Figure 11, the grain size in each sample is inhomogeneous, and the resistance to deformation differs for differently sized grains, thus a non-uniform fracture forms upon deformation. Another kind of non-uniform fracture morphology is caused by large pores in the facture as those shown in Figure 15e. As seen, the pore size is far greater than that of the dimple. The pore changes its shape during deformation, and because it breaks the matrix continuity, stress concentration occurs around it, leading to brittle fracture. As a consequence, the presence of large pores can seriously affect the tensile properties of the specimens.

## 4. Conclusions

(1) Thermal cycling has a decisive effect on the microstructure evolution of SLM low-alloy steel samples. The initial solidification microstructure of the molten pool is martensite. With the increase of EAD, martensite gradually transforms into a mixed microstructure of bainite and martensite–austenite, and into granular bainite in turn. Bainite ferrite gradually changes from lath to a multilateral structure under the action of subsequent thermal cycles.
(2) Thermal cycling also has a crucial effect on the grain size of the SLM low-alloy steel samples. The average grain size is 1.56 µm, 3.98 µm, and 6.31 µm at EADs of 142 J/mm^2^, 71 J/mm^2^, and 47 J/mm^2^, respectively. Thus, with the increase of EAD, the grain size is remarkably reduced due to the increase of austenitizing cycles and temperature.
(3) With the increase of EAD, yield strength and tensile strength of the low-alloy steel increase and their values far exceed those of the alloys prepared by traditional methods and those of alloy steels reported in literature. The change of elongation with EAD is not obvious.
(4) The grain size and microstructure are uneven in SLM low-alloy steel samples. The inhomogeneity becomes more significant with the decrease of EAD. When EAD is below 47 J/mm^2^, the fracture mechanism of the SLM low-alloy steel sample changes from ductile to a mixture of ductile and brittle fracture.

## Figures and Tables

**Table 1:** Chemical compositions of the low-alloy steel powder used in this work (wt %).

Element	C	Mn	Ni	Mo	Y	Fe
Content	0.15–0.25	0.6	1.0	0.5	0.01–0.05	Bal.

**Table 2:** Working parameters of the SLM process.

Layer Thickness (µm)	Laser Power (W)	Point Distance (µm)	Exposure Time (µs)	Scan Speed (mm/s)	EAD (J/mm^2^)
50	200	10	1000	10	284
50	200	10	500	20	142
50	200	10	250	40	71
50	200	10	166	60	47
50	200	10	125	80	36
50	200	10	100	100	29

EAD: energy area density.

**Table 3:** Thermal properties of SLM fabricated low-alloy steel at different temperatures.

Temperature (°C)	Density (Kg/m^3^)	Thermal Conductivity (W/m K)	Specific Heat (J/kg K)
25	7841	34.9	447
100	7820	33.4	450
300	7818	33.6	521
500	7827	30.4	615
700	7717	27.3	880
900	7609	27.0	609
1100	7504	29.5	639
1300	7399	31.9	673
1450	7347	33.2	696
1470	7193	33.3	1071
1510	7071	33.5	11,507

**Table 4:** Specific parameters of the thermal cycle process.

EAD (J/mm^2^)	Austenitizing Times	Maximum Heating Temperature (°C)	Heating Rate (°C/s)	Austenitizing Time (s)
142	7	1279	2.7 × 10^4^	0.03
71	4	1029	5.2 × 10^4^	0.008
47	2	868	10 × 10^4^	0.002

**Table 5:** Room temperature tensile properties of SLM low-alloy steel.

Sample Description	YS (MPa)	UTS (MPa)	EL (%)
EAD = 142 J/mm^2^	1256	1428	15.9
EAD = 71 J/mm^2^	1233	1385	16.2
EAD = 47 J/mm^2^	1205	1357	16.3
EAD = 36 J/mm^2^	982	765	5.8
Sample A	1080	1199	16.5
Wei [18]	956	1146	14.9
Zhouyue [17]	704.2	774.6	7.1
Tingting Guan [33]	702	901	15.2

YS: yield stress; UTS: ultimate tensile stress; EL: elongation.
